# Crossborder curriculum partnerships: medical students’ experiences on critical aspects

**DOI:** 10.1186/s12909-018-1239-6

**Published:** 2018-06-07

**Authors:** Dominique Waterval, Janneke M. Frambach, Stephen M. Scott, Erik W. Driessen, Albert J. J. A. Scherpbier

**Affiliations:** 1 School of Health professions Education, Maastricht University, P.O. Box 616, 6200 MD Maastricht, The Netherlands; 2 School of Health Professions Education, Faculty of Health, Medicine and Life Sciences, Maastricht University, Maastricht, the Netherlands; 3 Weill Cornell Medicine-Qatar, Qatar Foundation, Doha, Qatar; 4 Department of Educational Development and Research, Faculty of Health, Medicine and Life Sciences, Maastricht University, Maastricht, the Netherlands; 5 Faculty of Health, Medicine and Life Sciences, Maastricht University, Maastricht, the Netherlands

**Keywords:** Transnational education, Crossborder curriculum partnerschips, Internationalization, Student survey

## Abstract

**Background:**

The past decade has witnessed an upsurge in medical curriculum partnerships established across national boundaries to offer students at the foreign institution (host) a learning experience comparable to that of students at the exporting institution (home). However, since the learning environments and national healthcare contexts differ greatly between institutions, concerns have been raised in the literature about potential low quality of curriculum delivery, inadequate preparation of students to practice in the host country healthcare setting, and a culture shock for host students having to study a home curriculum.. The experiences and opinions of medical students related to these concerns have not been investigated. This study takes an explorative approach on key challenges faced by host institution students.

**Method:**

Three hundred sixty-one host students recruited from 3 partnerships completed a survey about their motives, transition from high school, language, preparedness for practice, future career planning, and general satisfaction. Descriptive statistics of closed-ended items and thematic analysis of open-ended items were performed.

**Results:**

Findings revealed that students generally held positive views of the education they received. Switching to a new language of instruction (English) and learning environment was not perceived as a major obstacle. However, a significant portion of students who as non-nationals did not speak the language of the patient population felt this complicated effective workplace-based learning.

**Conclusion:**

Despite differences in learning experiences, host students felt the partnership afforded opportunities to acquire unique academic competencies and boost their career. Further adaptation of the home curriculum to the host country healthcare system may be beneficial, without losing sight of medical curriculum partnerships’ potential to offer graduates an international outlook on global healthcare.

**Electronic supplementary material:**

The online version of this article (10.1186/s12909-018-1239-6) contains supplementary material, which is available to authorized users.

## Background

Worldwide, medical education institutions are establishing crossborder curriculum partnerships [1, 2]. In such partnerships, the curriculum, not students or faculty, crosses borders from the home location where it was developed to a host institution where it is delivered [3]. Host students expect a learning experience similar to that of their home institution counterparts. However, the learning environments of both institutions differ in terms of teachers, facilities, learning and other resources, and healthcare systems [4, 5]. Consequently, both student cohorts follow their curriculum in different learning and healthcare environments, potentially impacting the comparability of their learning experience.

As this new variety of internationalization is growing fast, so too the body of literature on this topic [6], albeit still preliminary [7], is expanding. Much attention is given to the effectiveness and desirability of this new form of internationalization based on an investigation of stakeholders’ motives and quality assurance frameworks [8]. However, few studies address the educational challenges crossborder curriculum partnerships face [9] and rarely base their conclusions on students’ voices as well.

Concerns have been raised that crossborder curriculum partnerships carry several risks which may impact students’ learning experiences, well-being, preparation for practice, and future career [10–12]. One potential risk is low quality of curriculum delivery. The literature has indeed reported instances of partnerships that have ended or delivered low-quality curricula, also in the medical domain [13]. In contrast, several quantitative survey studies report high levels of satisfaction among business and computer science students at Middle Eastern and Malaysian branch campuses [14–16]. Yet, students’ perceptions of crossborder curriculum partnerships in the medical domain were not included in these studies, and these partnerships face unique challenges related to international differences in healthcare systems.

A second risk is that host students, similar to exchange students, could experience “culture shock” when exposed to the new, foreign learning environment [10, 17, 18]. This shock may be caused by a switch to a non-native language of instruction (i.e., English) and to a student-oriented approach to learning [19–22]. Their secondary education may not have prepared them for the self-directed, student-centered methodologies that are characteristic of many exported medical curricula. Teachers and managers have indeed expressed concerns about the imposition of foreign approaches on host students [22]. This concern has furthermore been acknowledged by the British quality assurance agency for higher education in their adoption of guidelines that point out the need to consider “the cultural assumptions about higher education learning methods” [23]. A similar call has been made to international medical educators to be aware of ethnocentricity when exporting ideas and programs [24]. Unfortunately, little is known about the nature of this potential culture shock and its impact on host institution students.

A final risk is that the curriculum of the host institution does not adequately prepare students for practice in the *host* labor market, as it mirrors, albeit in a slightly adapted form, the curriculum of the home institution. A large survey outside the medical domain, for instance, noted that graduates of the host institution were highly skilled, but that their skills were not necessarily aligned with host country needs [25]. In such cases curriculum partnerships can be criticized for contributing too little to the host country context [26]. A related concern is the potential loss of human resources for host country healthcare if only a small proportion of graduates of these programs continue their training and professional career in the host country [27].

The aforementioned concerns have been recognized by the medical program directors responsible for the implementation and quality of six different medical curriculum partnerships [28]. Although they acknowledged that these were challenging issues that required continuous attention, they also believed that they generally did not interfere with the overall aims of the curriculum partnership. Strikingly, students’ views and experiences about these areas of concern were not solicited. This study explores host students’ perspectives on critical aspects of crossborder medical curriculum partnerships. Its outcomes may be useful for university leaders, branch campus managers and educators, policymakers, and other stakeholders. Together with our previous study involving medical program directors [28], this study contributes to a more comprehensive picture of this phenomenon. Furthermore, by concentrating on students as key stakeholders, this study can contribute to an in-depth understanding of issues and challenges in these partnerships.

## Methods

Our aim was to explore students’ experiences of challenges they face in crossborder curriculum partnerships, transcending a singular context. Therefore we used a self-administered survey consisting of a mix of open-ended and closed-ended items to investigate host students’ perceptions. As part of a larger research project, potentially eligible partnerships were identified using a snowballing technique: twelve independent international medical education experts were approached in person or by email, which yielded 22 potential partnerships. By means of an Internet research and email inquiry, this selection was further condensed to six partnerships that deliver a home institution’s curriculum at a host institution across borders and intend to provide comparable learning experiences to the geographically separated groups of students. Three institutions responded and participated; the remaining three declined due to a lack of time. Additional file 1 provides more details on these included partnerships. In all cases the initiative to establish a partnership originated from the host institution or country and served a domestic purpose. Furthermore, all host institutions offered a preparatory year which aimed to bridge the difference between secondary school and university with regards to content as well as language and study techniques. Host teachers were trained through mutual visits and online sessions about content and didactics.

### Research instrument

The survey consisted of 31 items which were drawn from a synthesis of literature on curriculum partnerships. Our search for an existing validated instrument that fulfilled our research objective and context was unsuccessful. We analyzed the articles included in two review studies on crossborder higher education [6, 9] for student-related issues. Six themes emerged from this analysis, specifically: motives, transition from high school, language, preparedness for practice, future career planning, and general satisfaction levels. We consequently structured the survey around these themes.

As we aimed to explore student experiences on all themes and offer them the opportunity to fully describe their experience, we used a mix of 5 Likert scale items, 8 closed-ended items, 11 full open-ended items and 7 structured open-ended items. The latter offered multiple answer options among pre-defined categories and provided the option “other” which students could use to articulate alternative views. The items and pre-defined answer options were based on the literature review. The structure was also designed to reduce survey fatigue.

Finally, five international students originating from the region in which data collection took place and for whom English was their second language pre-tested the survey for language and clarity. Program directors of the participating institutions also checked its face-validity and use of appropriate terminology. Their feedback led to further improvements. The final survey is included in Additional file 2.

### Data collection

Data were collected by using a convenience sampling procedure in order to reach as many host students as possible within each institution in all years of training. We initially set out an e-survey, but due to legal barriers (e.g. storing student data outside the host country) and low completion rate, we switched to a paper-based version. During on-site visits between December, 2015, and March, 2016 (starting period of the 2nd semester), the researcher or a research assistant selected accessible educational sessions in consultation with the host program director, inviting students to participate immediately after their session. Before distributing the survey, the researcher explained the study rationale and objectives, explicitly emphasized confidentiality, and asked participants to sign an informed consent form.

### Data analysis

We included all surveys that were completed until the final closed-ended item. This criterion led to the exclusion of two surveys, resulting in 361 completed surveys. However, response rates varied per item.

We performed descriptive statistics in SPSS of the closed-ended items and Likert scale items, which was supplemented with bivariate analysis of the variables “partnership,” “gender,” and “study phase,” wherever appropriate.

The responses to the full and structured open-ended items were analyzed thematically and categorized with the help of two research assistants. We developed an initial coding template based upon a first set of surveys. Through an iterative process codes were adjusted, refined or extended and all responses were categorized and until consensus was reached. The final categorization for each item was discussed and agreed upon by the research team. Disagreements were resolved by discussion. The results include the number and percentage of participants in case of single answer items and responses and percentage in case of multiple answer items.

## Results

### Demographics of respondents

Table 1 summarizes the demographic characteristics of our sample. We included a total of 361 surveys, representing nearly 50% of the total student population (740) at the three institutions.

**Table 1:** Demographic characteristics of the sample

	Partnership A	Partnership B	Partnership C	Total
Sample size	70	201	80	**361**
Population size	121	450	169	**740**
Gender				
No. of males (%)	70 (100)	83 (39)	30 (37)	**183 (51)**
No. of females (%)	0 (0)	128 (61)	50 (63)	**178 (49)**
Age				
Mean age (SD)	21.0 (2.5)	20.3 (1.8)	22.6 (2.1)	**21.0 (2.1)**
Study phase				
No. of pre-clinical students (%)	41 (59)	129 (61)	47 (59)	**217 (60)**
No. of clinical students (%)	29 (41)	82 (39)	33 (41)	**144 (40)**
Distribution of Nationalities				
No. of nationalities	11	6	24	**31**
No. of students with host-country nationality (%)	9 (13)	121 (57)	16 (20)	

The host student population in partnership A consisted exclusively of male students as they had no female students at that time; nonetheless, the total sample across the three institutions had an equal gender distribution (51% male, 49% female). To be able to explore how experience within the program influenced perceptions, we divided students according to their study phase. The ratio of pre-clinical (early years) to clinical students (later years) was approximately 60%:40% in all three institutions. The average age of the respondents was 21.

The host student population was heterogeneous in terms of ethnicity and background. We counted a total of 31 different nationalities. In partnership A, a relatively large proportion of respondents came from Syria (*N* = 30/69; 43%). The majority of students in partnership B were nationals of the host country (*N* = 121/211; 57%), closely followed by Malaysian students (*N* = 82/211; 39%). Partnership C had the most diverse mix of students, with nationals of the host country representing the largest proportion (*N* = 16/79; 20%). Based on analysis and grouping of the themes resulting from the explorative and qualitative data, we present our results clustered in two primary domains: the learning environment and the work environment.

### Learning environment: teaching method & program implementation

Students reported high levels of satisfaction with the study program across all partnerships and study years (Table 2). In general, respondents perceived their transition from high school to medical college as a shift to fewer contact hours and more freedom to determine the depth and range of their study. The majority (*N* = 231/355; 65%) reported they had overcome this transition to a new learning environment, and many (*N* = 169/208; 81%) indicated having reached this within one year or less. These findings were comparable across partnerships and by gender.

**Table 2:** General satisfaction levels; indicated on a 4-point Likert scale from very dissatisfied (1) to very satisfied (4)

	How satisfied are you with the study program?		
Institution	N	Mean (1–4)	Std. Deviation
Partnership A	65	2.72	.650
Partnership B	203	2.96	.566
Partnership C	78	3.14	.597
Total	346	2.95	.603

In commenting on the advantages of the program, many students (*N* = 192/361; 53%), especially those from later years, reported that the program, and in particular the student-centered teaching method, contributed to their academic and professional growth through added self-responsibility, confidence in public speaking, and collaboration with others. One student noted:

“*The system is really strong and I have already seen the difference between the students of my college and those of other colleges, regarding their level of education. […] In this system I have to be much more independent and do everything by myself. Right now this might be a little bit hard, but in the long run it will benefit me a lot.”*

*(1st-yr. student, Partnership A, item 28).*

Although students did not report major challenges, and their comments were generally constructive and positive, containing suggestions to improve the quality of the program implementation, some students were critical, and a few expressed frustration about the level of integration between the two institutions, for example:

*“It needs to be more integrated and the home institution should be more aware of the host institution” (4th-yr. student, Partnership C, item 31).*

When asked explicitly about the perceived disadvantages for their learning experience compared to their home counterparts, many students (*N* = 161/361; 45%) identified differences in learning facilities, differences in the conduct of educational sessions, and staff being perceived as less qualified to conduct sessions. The emphasis of these comments varied across institutions. One student described that

“They have more access to libraries, electronic books and websites; they have better health institutions and training; they have more updated knowledge and more wide-minded doctors.” (5th-yr. student, Partnership B, item 30)*.*

### Learning environment: language

A second issue regarding the learning environment of host students was the use of English as the principal language of instruction. For the vast majority of respondents English was their second language (339/361; 94%). However, most of them did not consider this as a major impediment to their study (*N* = 317/355; 90%). Approximately one third of respondents (*N* = 102/361; 28%) reported that additional activities were needed to address language deficiencies, such as online language courses and deliberate practice with friends and family. A substantial group (*N* = 148/343; 43%) indicated some language problems, mostly when answering test items, in group discussions, self-study and in understanding teachers for whom English was also a second language:

“The teachers have an excellent command of English, but some have accents that need some time getting used to. English is not used at all in interactions with patients. Since I have a limited command of Arabic, I have limited interactions with patients.” (6th-yr. student, Partnership B, item 21)*.*

This comment addresses another language issue which emerged from our data analysis. Due to the international student intake at the host institutions, a large proportion of respondents did not speak the primary language of the host institutions’ patient population. As a result, students faced challenges when interacting with patients, especially in the clinical phase of the curriculum. This influenced the learning experiences of many respondents (115/216; 53%) and was mentioned comparably across institutions. Some respondents suggested pragmatic strategies for overcoming the language barrier, such as using student or professional translators, or learning the host country’s language. Some reflected that learning to deal with the inability to communicate directly with patients is a valuable skill:

“There is diversity which already prepares me for the diversity of healthcare in the workplace. Interactions are more meaningful when people come from different cultures.” (1^st^-yr. student, Partnership C, item 30)*.*

### Work environment: match with host country healthcare system

Another issue was how students perceived their preparedness for practice in the host country healthcare context. Respondents had already garnered substantial experience in the host country healthcare system: the pre-clinical students had made short visits and assignments in the host country healthcare setting, while the clinical students had done rotations. Overall, most students felt either appropriately trained to work in the host country healthcare system or they had a neutral opinion about it (Table 3).

**Table 3:** Preparedness for practice on a 5-point Likert scale from very inappropriate (1) to very appropriate (5)

	How do you feel your current study program prepares you for your experience in the host country healthcare setting?		
Institution	N	Mean (1–5)	Std. Deviation
Partnership A	70	3.29	.965
Partnership B	207	3.33	.824
Partnership C	79	3.96	.688
Totals	356	3.46	.866

When asked whether they had missed important topics in their study program in relation to the host country healthcare context, half of the students replied in the affirmative (*N* = 150/361; 42%). There was strong inter-institutional variation in the curriculum elements that were perceived as missing or not fully implemented. However, students (*N* = 46/150; 31%) in all institutions expressed the need for more information about the host country’s healthcare and legal system, ethical values, and appropriate behavior. For example,

*“The health system of the host country requires an understanding of many different cultures as well as of languages, issues which I feel weren’t particularly addressed although we did have lectures about these issues, we still face problems of this kind in the local (host) health setting.” (1st-yr. student, Partnership C, item 12).*

This suggests that while theoretical lectures about the home or host context may be helpful, they may not sufficiently address language and culturally appropriate clinical behaviors.

A smaller proportion of students (*N* = 85/348; 24%) perceived certain curriculum content as irrelevant to the host healthcare setting. Their remarks concentrated on information and data that seemed relevant only in the home country, such as epidemiological, social and cultural aspects, and facts on certain topics that were not applicable to their host contexts. For example:

*“We learn about the prevalence of diabetes in the home country, with its geographical distribution. I do not believe it is relevant to study this in our setting which is thousands of miles away. Most of the graduates will practice medicine in this country and not in the home [country]. Consequently, I think we should learn more about here. It’s good to learn about the situations in other countries but not in such detail.” (1st-yr. student, Partnership A, item 12).*

At the same time, however, students also highlighted the potential benefits of acquiring more knowledge about the home country, as the following quote illustrates:

*“Being in the host country and taking a home program, there are some things in a cultural perspective that don’t relate so much to our society ... However, studying medical conditions that are more relatable to the home country can help us to learn about it if we want to work there in the future.” (5th-yr. student, Partnership B, item 14).*

This revealed a two-sided picture: While some students, logically, did not like being exposed to blunt copying and pasting of irrelevant curriculum materials, others did value learning about professional practice and diseases in the home country, as this broadened their perspective.

### Work environment: future career path

Students seemed very aware of their unique profile and potential career paths. Many students (*N* = 192/361; 53%) mentioned that studying as a host student in their partnership would bring specific advantages to their future careers, such as more academic and professional growth. A few were able to compare their education with that of conventionally trained friends and expressed confidence in their own level of training. 87 of these 192 students felt that an internationally oriented profile was an explicit advantage:

*“[…] it will make me a good doctor and help me to travel abroad in order to do some more research on medicine. It will help me to communicate with other best doctors from all over the world and to gain some of their experience.” (2nd-yr. student, Partnership B, item 28).*

For many, the international profile of the institution was a specific reason to choose the program. Although students reported several other reasons, many were attracted by the international reputation of the home institution (170/357; 48%) and by the prospect of continuing or spending part of their study at the home institution. Nearly all participants were aiming for specialty training as the next step in their medical careers. The UK and the US (*N* = 81/269;23% and *N* = 104/360; 29%, respectively) were cited as the leading target destinations, followed by Asia (*N* = 50/360; 14%), the Middle East (*N* = 35/360; 10%), and Europe (*N* = 29/360; 8%).

Table 4 lists for each partnership the proportion of students that indicated a desire to pursue studies in the student’s country of origin, home institution’s country, host institution’s country, or other country, respectively. It should be noted that the categories overlap, hence we should be cautious while making inferences. While a large proportion of students in partnership C indicated interest in training in the country of the home institution, students in the other partnerships were more divided. The data showed that a relatively small proportion of students (*N* = 25/241; 10%) had intentions to stay in the host country for postgraduate training; they either wanted to return to their country of birth or had international ambitions.

**Table 4:** Country of destination after graduation

		Country of destination after graduation				Total
		Country of birth/ethnicity	Country of home institution	Country of host institution	Other	
Partnership A	Count	3	3	7	15	28
	% within institution	10.7%	10.7%	25.0%	53.6%	
Partnership B	Count	56	66	7	47	176
	% within institution	31.8%	37.5%	4.0%	26.7%	
Partnership C	Count	7	63	11	4	85
	% within institution	8.2%	74.1%	12.9%	4.7%	
Total	Count	66	132	25	66	289
	% of total	22.8%	45.7%	8.7%	22.8%	100.0%

A number of students (*N* = 63/161; 39%) shared concerns that they would have difficulty securing postgraduate training or other positions in their future work context. They believed that they might not be considered “good-quality graduates” with a worthy degree, due to the lack of a positive or established reputation of graduates from international medical programs. One pre-clinical student noted that

“[it will be] harder to find a residency program and it [will be] difficult for all programs to be aware of who we are.” (2^nd^-yr. student, Partnership C, item 28)

Although most of them were positive about the quality of their training, they felt a sense of “distrust” of the outside world. As these partnerships are relatively young, many partnerships do not have a body of alumni to ‘promote’ the host institution. Even in partnership B, which has existed since 2006, students felt that the relatively young program, despite its collaboration with a reputational foreign institution, still faced suspicion within the host country.

## Discussion

This study sought to explore medical host students’ perceptions of a number of educational concerns raised in the literature about cross-border curriculum partnerships. Students’ overall levels of satisfaction were high and comparable to those reported outside the medical domain [14–16, 29]. Students particularly valued the home program for its student-centered teaching method, its international profile, and higher career prospects compared to local alternatives available to them. This latter observation is an important factor for students who are unable to leave their home region or have no intention to do so [30]. Moreover, students shared medical program directors’ view [28] that most of them needed only a few months to adapt to English as the main language of instruction and to student-centered education. Some students even flagged these two aspects as clear advantages. Although students did identify many areas for improvement, they also realized that exact similarity of their learning experience compared with home students would be unattainable, as Coleman was already keen to point out [31]. In all, this study seems to suggest that although an alleged “culture shock” [10, 17, 18] could indeed be observed in these medical curriculum partnerships, its consequences were of manageable magnitude.

Nevertheless, our findings do to some extent confirm the validity of concerns about students’ preparedness for practice [11, 20]. The fact that host institutions attracted students from different nationalities, for instance, created situations in which some of the host students did not master the language of the host country’s patient population. Such hurdles represent a serious threat not only to deliver comparable quality but also to prepare students to work in the host context. Potential ways for institutions to anticipate such problems, for instance, are to include compulsory host-language instruction in the pre-clinical years or to organize a binary clinical placement system whereby students who speak the host language see host country patients, and those who do not encounter English-speaking patients [14–16, 28–31].

A fundamental issue to students’ preparedness for practice is the observation that students often pursue their professional careers in contexts other than the ones they were trained in. Our study’s student population had diverse career plans and international ambitions, while their planned destinations differed across partnerships. This leads to the question: is the partnership’s aim to train students for the local context, home context or to prepare them for an international career? The answer to this question will affect decisions about curriculum content, implementation, and delivery, determining how and to what extent the home program should be adapted to the context of the host institution, and ultimately shaping students’ learning experiences and preparedness for their desired practice.

Answering this question, however, is no easy feat, as ethical issues should be considered. The WHO, for instance, urges medical institutions to be socially accountable to their host country contexts and to address the needs of the host population [32]. In a similar vein, scholars have warned against a copy-paste approach to curriculum partnerships, stressing the need to adapt the home program to the host context as much as possible [26, 31]. Yet, we have seen that students often aspire to future careers outside the host country healthcare setting, hence an overemphasis on curriculum adaptations to fit the host context may not be in their best interest. Not only the respondents in this survey voiced this concern, but also host students who were interviewed in depth elsewhere did so [29]. These considerations would support an adaptation of the home institution’s curriculum to make it more globally oriented.

It can be argued that international curriculum partnerships are in a unique position to offer such globally oriented learning experiences. Upon entering the program, host students are immersed in an international learning community and are taught an (adapted) foreign medical curriculum by teachers from different countries. In comparison to their home counterparts, host students have a more heterogeneous background [18], as was the case in our study. The healthcare system they are trained in is different from the system in their country of origin and destination. These features offer opportunities to develop students’ adaptability to new working contexts and to colleagues and patients with diverse backgrounds, which might be an essential attribute of internationally oriented health professionals, and perhaps even of health professionals in any context. The challenge for medical educators lies in framing and positioning the diversity that exists within these programs in a way that is meaningful and attractive to students, while simultaneously doing justice to the host context.

Students in this study mentioned they missed important host country healthcare-related topics in their program and felt certain topics were irrelevant to the host context, although they did appreciate the benefits of the latter for their future international career. Efforts to cultivate adaptability by integrating host country and international topics into the curriculum can yield meaningful learning activities for a wide range of students. An example could be to engage medical students in host context-related patient and physician narratives and have them reflect on the implications for professional practice in both the host context and elsewhere [33]. By engaging students and possibly other relevant stakeholders in meaningful locally prioritized issues, such an approach would also allow institutions to become more socially accountable to their host contexts.

If partnerships seize the opportunity to distinguish themselves this way, they might establish a reputation as institutions for global-local learning in medical education, rather than being viewed with suspicion by other players in the field, something the students in this study feared. Future research could explore these possible avenues of adaptation and their implications, including the perceptions of graduates of these programs a number of years after graduation to identify their career paths and the role of the curriculum partnership.

The aim of this study was neither to assess the quality of the learning experience of host students nor to make any judgements on particular partnerships. This would require more in depth case-specific investigation, as well as involving other stakeholders, such as teachers. Questions that could be asked are for example: How do teachers work with the materials coming from the home institution?

This study is part of a larger research project in which data from different stakeholders, i.e. medical programme directors [28], host medical students (this study), host medical teachers [34], a case study from a home perspective [35], and a literature review including insights from outside the medical domain [9] are collected. Together they form a comprehensive picture of this complex and challenging form of internationalization.

One of this study’s merits is that it garnered the perceptions of a rather unexplored group of stakeholders in this new form of internationalization in medical education. Their comments provide meaningful insights into the way host students attend to concerns voiced in the academic field. It should be noted, however, that our conclusions are explorative as the survey instrument has not been validated in an international setting and its items may be subject to different interpretations by participants. Notwithstanding, we feel the survey construct and opportunity to ask questions about the content while completing the survey ensures that the instrument fits the explorative aim of the study.

Another limitation of this study is that all partnerships’ host institutions were located in the same region, the Middle East. Since the cultural norms and nature of the local healthcare setting in this region undoubtedly affected host students’ responses, the findings cannot be automatically transferred to other contexts. Finally, the partnerships we included varied in their setup and collaboration intensity, potentially influencing the results, which we attempted to remedy by concentrating our analysis on the overarching themes and perceptions. We see this survey as a first step in exploring the experiences and challenges faced by students in medical curriculum partnerships. Further research could address particular topics in this survey in greater detail such as the cultural and ethical aspects of implementing curricula across contexts.

## Conclusion

Host students felt that medical curriculum partnerships offered a valuable learning experience. Despite organizational differences between partnerships, institutions shared several similarities in terms of host students’ perceptions of the quality of their education, transition to student-centered education, learning in a second language, and match between the home curriculum and the host country healthcare system. Yet, the international mix of students posed additional challenges such as the language barrier between students and the patient population available for clinical training, which called for specific and timely remediation measures. Medical curriculum partnerships that capitalize on the international learning environment of host institutions and create a meaningful synergy between globally and locally oriented adaptations may contribute to the wide spectrum of medical graduates and doctors needed worldwide.

## Additional files

Additional file 1: Included partnerships: An overview of the home and host country of the partnerships, type of programme, start of the first batch, main methods of instruction. (DOCX 16 kb)

Additional file 2: Survey: Complete overview of the survey given to the participants, including informed consent and explanation. (DOCX 35 kb)
