# Supplementary material for: Crossborder curriculum partnerships: medical students’ experiences on critical aspects
Source: BMC Med Educ. 2018 Jun 7;18:129. doi: 10.1186/s12909-018-1239-6 (PMC5992638; doi:10.1186/s12909-018-1239-6)
Supplement: Supplementary file 1 — Included partnerships: An overview of the home and host country of the partnerships, type of programme, start of the first batch, main methods of instruction. (DOCX 16 kb) [file 12909_2018_1239_MOESM1_ESM.docx]

## Additional file 1: Included partnerships

Table 1: Partnerships’ codes and characteristics

| **Partnership’s code** | **Country of home institution** | **Country of host institution** | **Type of program** | **Start first batch** | **Main methods of instruction** |
| --- | --- | --- | --- | --- | --- |
| **A** | The Netherlands | Saudi Arabia | 6-year undergraduate | 2010 | PBL and Lectures |
| **B** | United Kingdom | Egypt | 6-year undergraduate | 2006 | PBL and Lectures |
| **C** | United States | Qatar | 4-year postgraduate | 2002 | PBL and Lectures |
