# Supplementary material for: Crossborder curriculum partnerships: medical students’ experiences on critical aspects
Source: BMC Med Educ. 2018 Jun 7;18:129. doi: 10.1186/s12909-018-1239-6 (PMC5992638; doi:10.1186/s12909-018-1239-6)
Supplement: Supplementary file 2 — Survey: Complete overview of the survey given to the participants, including informed consent and explanation. (DOCX 35 kb) [file 12909_2018_1239_MOESM2_ESM.docx]

## Additional file 2: Survey

Students' perceptions of crossborder curriculum partnerships

Dear student,

We would like to invite you to participate in this survey on "Students’ perceptions of crossborder curriculum partnerships" in the medical domain. As you are aware, the medical program that you are following is the result of a curriculum partnership between your institution and a foreign institution in the West. The goal of our research is to better understand these medical partnerships and develop implementation guidelines. Therefore we are eliciting perceptions and experiences from various medical schools around the world. Your perspectives are therefore of great value to the scientific community. We would like to emphasize that this survey is **not** a quality review or evaluation of the program that you are following.

**Terminology**

In this survey, the foreign partner university is called the “home institution,” and its students are called “home students.” Your own university is called the “host institution” and you are a “host student.”

**Type of Research Intervention**

This research will involve your participation in a paper-based survey consisting of closed- and open-ended questions. The survey will take about 20 minutes to complete.

**Participant Selection**

You are being invited to take part in this research because you are a registered student among one of the 6 institutions worldwide that are participating in our research. All of these students are being invited to share their perspectives.

**Confidentiality**

Participation is strictly anonymous, and we will not ask any personal information. You are invited to leave your email address at the end in case you would like to be informed about the final results, but this is voluntary. The data will be stored according to the rules of the School of Health Professions Education, Maastricht University, for a period of 5 years and will only be accessible by the research team.

**Risks**

Besides the time investment, there are no reasonable foreseeable risks or discomforts to participate.

**Benefits**

There will be no direct benefit to you, but your participation is likely to help us find out more about how to create sustainable crossborder curriculum partnerships that ensure a similar learning experience to students at both home and host institutions.

**Right to Refuse or Withdraw**

Refusal to participate and/or discontinuity will involve no penalty or loss of benefits in any way.

This research proposal has been reviewed and approved by the Ethical Review Board of the NVMO (Netherlands Association for Medical Education) If you wish to find out more about the NVMO, contact Ms. M. Sterman, secretariaat@nvmo.nl, telephone: +31 (0)88 755 9911.

**Who to Contact**

XXX

**Informed Consent**

I have read the information for participant in the introduction to the survey. I was able to ask the researchers additional questions. My questions were adequately answered. I had sufficient time to decide whether to participate.

I am aware that participation is completely voluntary. I know I can quit at any particular moment in time without providing a reason and without incurring any penalty or loss.

I know that the research team mentioned on the introduction page of the survey has access to the data.

I consent to the use of my data for the purpose mentioned in the introduction page of the survey.

I understand the rationale and setup of this survey.

Therefore, I agree to participate in this survey.

- Yes
- No (please don’t proceed with the survey unless you can state yes.)

**THE FOLLOWING ITEMS TELL US ABOUT YOU:**

1. What is your gender?

- Male
- Female

2. What is your age?

_______________________

3. What is your nationality?

_______________________________

4. In which institution are you following your medical study program?

- Partnership A
- Partnership B
- Partnership C

5. In which year did you start this study program?

- 2009
- 2010
- 2011
- 2012
- 2013
- 2014
- 2015
- Other, please specify ____________________

6. Why did you choose this study program?

(you can tick multiple answers)

- Because I wanted to continue my studies at this specific partnering (home) institution
- Because I satisfied the entry criteria
- Because of the international reputation of the (home) study program
- Because of the PBL system
- Because of the proximity of my institution
- Other, please specify ____________________

7. Which year are you currently in? (Please count from when you entered the educational program at your institution.)

- 1st year (i.e., premedical curriculum year 1)
- 2nd year
- 3rd year (i.e., medical curriculum year 1)
- 4th year
- 5th year
- 6th year
- Preparatory year

**THE FOLLOWING ITEMS TELL US MORE ABOUT THE MATCH BETWEEN YOUR STUDY PROGRAM AND LOCAL HEALTHCARE SYSTEM**

8. In your study program, what experience did you have of the local healthcare setting (meaning the healthcare system of the country you are currently in – the host country)?

You may tick multiple answers:

- I have experience because I worked in the local healthcare system before
- I did some rotations/internships
- I have made visits to healthcare centers
- I have made (short) assignment(s) that took place in the local healthcare setting: for example interviewing patients
- Other, please specify ____________________

9. How much time have you spent on average in the local professional healthcare setting? Please write your answer in the following format:

No. hours per week for no. months.

______________________________________________

________________________________________________

10. How do you feel your current study program prepares you for your experience in the local healthcare system?

(please select the option that most applies to your situation)

|  | Very Inappropriately | Inappropriately | Neutral | Appropriately | Very Appropriately |
| --- | --- | --- | --- | --- | --- |
| I feel it prepares me |  |  |  |  |  |

11. When thinking about your experiences in your healthcare setting, is there any topic (e.g., medical knowledge, skills, or attitudes) in the study program that you missed because in your experience it was needed in the host (your) healthcare setting?

- Yes
- No

12. If yes, please indicate which topics, i.e., knowledge, skills or attitudes, that were not covered during the study program but according to your experiences are relevant in the host (your) healthcare setting;

__________________________________________________________________________________________________________________________________________________________________________________________________________________________________________________________________________________________________________________________________________________________________________________

13. Do you think that you have studied topics (medical knowledge, skills, or attitudes) in the (home)  program that are not relevant in the host healthcare setting?

- Yes
- No

14. If yes, please indicate which topics, i.e., knowledge, skills, or attitudes, that are included in the study program but according to your experiences are not relevant in the host healthcare setting:

__________________________________________________________________________________________________________________________________________________________________________________________________________________________________________________________________________________________________________________________________________________________________

__________________________________________________________________________

**THE FOLLOWING ITEMS TELL US MORE ABOUT YOUR TRANSITION FROM HIGH SCHOOL TO UNIVERSITY**

15. How would you describe the amount of contact hours and freedom to determine depth and range of study in your high school experience and in your current study program?

(please select the option that most applies in each situation)

|  | A lot of contact hours and no freedom to determine depth and range of study | Many contact hours and a little bit of freedom to determine depth and range of study | Little contact hours and some freedom to determine depth and range of study | Few contact hours and a lot of freedom to determine depth and range of study |
| --- | --- | --- | --- | --- |
| High school |  |  |  |  |
| Current program |  |  |  |  |

16. Do you feel you have overcome this transition (between your high school experience and your current study program)?

- Yes
- No
- Not sure

17. If yes, how much time did it approximately take you to overcome this transition?

______________________________________________________________________________________________________________________

__________________________________________________________________________

18. What kind of activities helped you to cope with the transition to your current academic program? (you may tick multiple answers)

- Individual coaching and mentoring
- Studying by myself
- Study skill courses
- No specific activities
- Other, please specify ____________________

**THE FOLLOWING ITEMS TELL US MORE ABOUT LANGUAGE ISSUES**

19. Please indicate whether English is your:

- Mother tongue
- Second language
- Other, please specify ____________________

20. Do you find it difficult to study medicine in English? (please select the option that best applies to you)

|  | Strongly Disagree | Disagree | Agree | Strongly Agree |
| --- | --- | --- | --- | --- |
| I find it difficult to study medicine in English |  |  |  |  |

21. At this point of time in my studies, my competency in English language is hindering the level and depth of my:

(you can tick multiple answers)

- Answering of test questions
- Discussion in group sessions
- Interaction with patients
- Self-study
- Understanding of teachers
- None
- Other, please specify ____________________

22. Do you feel that your proficiency in English has a negative impact on the quality of your learning experience? (please select the option that best applies to you)

|  | Strongly Disagree | Disagree | Neither Agree nor Disagree | Agree | Strongly Agree |
| --- | --- | --- | --- | --- | --- |
| My English language skills have a negative impact on the quality of my learning experience |  |  |  |  |  |

23 Have you undertaken any activities to overcome language difficulties?

- Yes
- No

24. If yes, please specify which activities, and what has been the effect of these activities?

___________________________________________________________

___________________________________________________________

___________________________________________________________

25. Which language is spoken by the majority of the patients you encounter? How does this affect communication?

___________________________________________________________________________________________________________________________________________________________________________________________________________________________________________

**THE FOLLOWING ITEMS TELL US MORE ABOUT YOUR FUTURE CAREER**

26. What are your plans after graduation?

- Apply for specialty training
- Do not know yet
- Other, please specify ____________________

27. In which country are you planning to apply for specialty training?

- UK
- USA
- Host country I am living in now
- Country of birth
- Other country, namely ____________________
- No idea yet

28. Do you feel that there are specific advantages and/or disadvantages for finding and carrying out your future job, due to the fact you will graduate from a crossborder medical study program? Please specify the advantages and/or disadvantages.

___________________________________________________________

___________________________________________________________

___________________________________________________________

___________________________________________________________

___________________________________________________________

___________________________________________________________

___________________________________________________________

___________________________________________________________

___________________________________________________________

**THE FOLLOWING ITEMS TELL US MORE ABOUT OTHER ISSUES**

29. How satisfied are you with the study program?

|  | Very Dissatisfied | Dissatisfied | Satisfied | Very Satisfied |
| --- | --- | --- | --- | --- |
| How satisfied are you with the study program? |  |  |  |  |

30. In which way do you think your learning experience differs from the learning experience of home students? (i.e., students of the partnering institution)

___________________________________________________________

___________________________________________________________

___________________________________________________________

___________________________________________________________

___________________________________________________________

___________________________________________________________

31. Do you have any other remarks about this survey or your experiences with your crossborder medical study program?

___________________________________________________________

___________________________________________________________

___________________________________________________________

___________________________________________________________

___________________________________________________________

___________________________________________________________

___________________________________________________________

___________________________________________________________

___________________________________________________________

**Thank you for your participation!**

We would be pleased to send you a summary of the survey results. If you wish to receive this summary, please state your email address. Your email address will only be used for this purpose and will be treated confidentially. For any questions feel free to contact: d.waterval@maastrichtuniversity.nl

___________________________________________________________
